# Multiplex-Heterogeneous Network-Based Capturing Potential SNP “Switches” of Pathways Associating With Diverse Disease Characteristics of Asthma

**DOI:** 10.3389/fcell.2021.744932

**Published:** 2021-12-14

**Authors:** Ming-Yu Ran, Zhang Yuan, Chui-Ting Fan, Zhou Ke, Xin-Xing Wang, Jia-Yuan Sun, Dong-Ju Su

**Affiliations:** ^1^ Department of College of Bioinformatics Science and Technology, Harbin Medical University, Harbin, China; ^2^ Department of Respiratory, The Second Affiliated Hospital of Harbin Medical University, Harbin, China; ^3^ Shanghai Chest Hospital, Shanghai Jiaotong University, Shanghai, China

**Keywords:** bioinformatics, asthma, ceRNA, pathway enrichment analysis, miRNA, lncRNA, network, SNPs

## Abstract

Asthma is a complex heterogeneous respiratory disorder. In recent years nubbly regions of the role of genetic variants and transcriptome including mRNAs, microRNAs, and long non-coding RNAs in the pathogenesis of asthma have been separately excavated and reported. However, how to systematically integrate and decode this scattered information remains unclear. Further exploration would improve understanding of the internal communication of asthma. To excavate new insights into the pathogenesis of asthma, we ascertained three asthma characteristics according to reviews, airway inflammation, airway hyperresponsiveness, and airway remodeling. We manually created a contemporary catalog of corresponding risk transcriptome, including mRNAs, miRNAs, and lncRNAs. MIMP is a multiplex-heterogeneous networks-based approach, measuring the relevance of disease characteristics to the pathway by examining the similarity between the determined vectors of risk transcriptome and pathways in the same low-dimensional vector space. It was developed to enable a more concentrated and in-depth exploration of potential pathways. We integrated experimentally validated competing endogenous RNA regulatory information and the SNPs with significant pathways into the ceRNA-mediated SNP switching pathway network (CSSPN) to analyze ceRNA regulation of pathways and the role of SNP in these dysfunctions. We discovered 11 crucial ceRNA regulations concerning asthma disease feature pathway and propose a potential mechanism of ceRNA regulatory SNP → gene → pathway → disease feature effecting asthma pathogenesis, especially for MALAT1 (rs765499057/rs764699354/rs189435941) → hsa-miR-155 → IL13 (rs201185816/rs1000978586/rs202101165) → Interleukin-4 and Interleukin-13 signaling → inflammation/airway remodeling and MALAT1 (rs765499057/rs764699354/rs189435941) → hsa-miR-155 → IL17RB (rs948046241) → Interleukin-17 signaling (airway remodeling)/Cytokine-cytokine receptor interaction (inflammation). This study showed a systematic and propagable workflow for capturing the potential SNP “switch” of asthma through text and database mining and provides further information on the pathogenesis of asthma.

## 1 Introduction

Asthma is a chronic inflammatory disease of the airways. It is a physiological hyperreactivity that causes recurrent wheezing, chest tightness, or coughing in patients. The contribution of genetic variants, especially single nucleotide polymorphisms (SNPs) to asthma has become the subject of widespread research and gratifying results have been achieved in some independent studies. A case-control study containing 96 asthma children and 86 healthy children showed that ADAM33 rs678881 polymorphism is significantly correlated with increased susceptibility to asthma in Chinese Han children (Ning et al., 2019).

Long non-coding RNAs (lncRNA), a type of RNA defined as non-protein coding transcripts with a length exceeding 200 nucleotides, is universally acknowledged to be the main participant of functional ceRNA mechanism. MicroRNA (miRNA), a 20–22 nucleotide conserved non-coding RNA molecule that functions in RNA silencing and the post-transcriptional regulation of gene expression by targeting the 3′ untranslated region (3′UTR) of specific messenger RNAs (mRNAs) for degradation or translational repression, has been shown to participate in varieties of biology progresses such as signaling transduction pathways, cell cycle, differentiation, and apoptosis. The significant roles of lncRNAs and miRNAs in the pathogenesis of asthma have been broadly demonstrated in many studies. The distinct change of expression of lncRNA PVT1 was observed in patients with corticosteroid-sensitive severe asthma. Subsequent targeting studies in ASMCs from patients with severe asthma showed that inhibition of lncRNA PVT1 with siRNAs increases FCS plus TGF-β–induced cellular proliferation through targeting of the transcription factor c-MYC, revealing its importance in controlling both proliferation and IL-6 release in ASMCs (Austin et al., 2017). MicroRNA 638 is a primate-specific miRNA that plays crucial roles in development, DNA damage repair, hematopoiesis, and tumorigenesis. Researchers have found that the expression of miR-638 is significantly downregulated in proliferative human ASM cells in response to various mitogenic stimuli, including PDGF-BB, FBS, and TGF-β1. Adenovirus-mediated miR-638 overexpression markedly inhibits ASMC proliferation and migration, while ablation of miR-638 by anti-miR-638 increases cell proliferation and migration. A series of experiments have demonstrated that miR-638 overexpression significantly reduces the expression of downstream targets cyclin D1 and NOR1, both of which have been proven to be essential in cell proliferation and migration (Wang et al., 2018).

In molecular biology, competing endogenous RNAs (abbreviated ceRNAs) regulate other RNA transcripts by competing for shared microRNAs (miRNAs), and new mechanisms of interaction between RNAs have been revealed. LncRNA-miRNA-mRNA regulation is one such example. LncRNA molecules sequester shared microRNAs (miRNAs), thereby affecting the expression of other targets of the miRNAs. Individual ceRNA mechanisms have been broadly researched in asthma. Treg/Th17 imbalance plays an essential role in the pathogenesis of asthma. A study found that alternation of LncRNA-MEG3 can change the function and result in increasing the percentage of Th17. Subsequent experiments indicate that LncRNA-MEG3 could inhibit the level of microRNA-17 as a competing endogenous RNA (ceRNA) and the microRNA-17 suppressed Th17 though targeting RORγt directly (Qiu et al., 2019). The sequence complementarity and thermodynamics of miRNA-lncRNA and miRNA-mRNA binding play crucial roles in their interactions. Hence, it is conceivable that lncRNA-associated or miRNA-associated single nucleotide polymorphisms (lncSNPs or miRSNPs) could influence the regulation of lncRNAs or miRNAs (Wilk and Braun, 2018; Gebert et al., 2020).

LncSNPs located in lncRNA genes could influence the biogenesis of lncRNA. The lncSNPs located in the miRNA binding sites could also alter the structure to affect the function of binding miRNA and render them the primary causative genetic variant. MiRSNPs have been divided into two categories depending on their locations, i.e., SNPs within the miRNA target site and SNPs within miRNA genes. SNPs within miRNAs genes could influence all states of miRNAs synthesis (pri-, pre-, and mature) and alter the expression level or function of miRNAs. SNPs within miRNA target sites may undermine, weaken or produce the interactions between miRNA and miRNA target and issue in a corresponding decrease or increase in protein translation. In bladder cancer, the lncRNA TINCR rs2288947 A > G variation was associated with increased expression of lncRNA TINCR in cancer tissues, and the rs8113645 C  >  T was associated with decreased expression (Xu et al., 2021).

The biological pathway, which comprises a cluster of interacting genes, is a useful construct for gaining insight into the underlying biological mechanism of genes and proteins. Reducing complexity and increasing explanatory power are the advantages of analysis at the pathway level (Khatri et al., 2012). Therefore, disease pathogenesis analysis in terms of the pathway is a widely used method in many studies. As mentioned above, lncRNA and miRNA play significant roles in regulating gene expression as post-transcriptional regulators. Thus, the weakness or destruction of lncRNA-miRNA or miRNA-mRNA binding could exert a tremendous influence on target-associated pathway regular functions.

Recently, a case-control study evaluated the association of SNP (rs140618127) in lncRNA LOC146880 with non-small cell lung cancer (NSCLC) in 2,707 individuals. Lab experiments *in vitro* and *in vivo*, which were designed to explore the mechanism of the SNP’s biologic influence, indicate that SNP rs140618127 contained a binding site for miR-539-5p, and that the binding between miR-539-5p and LOC146880 results in declined phosphorylation of an oncogene, ENO1. The reduced phosphorylation of ENO1 leads to decreased phosphorylation of the PI3K and Akt pathways, which is further linked to a decline in cell proliferation and tumor progression (Feng et al., 2020). Nevertheless, to date, little research has elaborated the effects of lncSNPs or miRSNPs in asthma. The present study aimed to construct a comprehensive catalog of the typical features of asthma-associated lncSNPs and miRSNPs and analyze the potential SNP-mediated dysfunctions of disease feature pathways.

This study generates new insights into the pathogenesis of asthma by integrating disease characteristics, pathways, and SNPs information. Three typical disease characteristics were scrupulously determined according to the summary of asthma characteristics in classic retrospective articles (Papi et al., 2018; Zhu et al., 2020b), i.e., airway inflammation, airway hyperresponsiveness, and airway remodeling, etc. A contemporary catalog of corresponding risk transcriptome including mRNAs, miRNAs, and lncRNAs was manually established. This contributes to analyses of specific parts and the overlapping characteristics of asthma disease in terms of gene function and related pathways and a more detailed and comprehensive understanding of the pathogenesis of asthma. In consideration of the limitations of the current method of pathway identification when thoroughly applying to our gathered information, a novel algorithm MIMP was developed, which accurately and comprehensively identifies pathways while exploiting suboptimal networks and can take account of current and closely pathway-associated transcriptomics and proteomics information. The MMIP can be used to identify the pathway association for a disease feature-related blended miRNA-mRNA molecular set. We then construct a ceRNA network for each recognized disease feature pathway and systematically identify candidate functional lncSNPs and miRSNPs and their potential mechanisms on the pathway, based on current genetic findings for asthma, which can help to further elucidate their potential roles in the pathogenesis of asthma both in genetic variant and at the post-transcriptional regulation level.

## 2 Materials and Methods

### 2.1 Human Asthma Risk Transcriptome Data Collection

Asthma risk transcriptome was defined for biomolecules including the mRNA, miRNA, and lncRNA involved in the pathogenesis of asthma, rather than simply showing differential expression in asthma. We examined literature on the features of asthma disease published before March 3, 2021, by searching PubMed (https://pubmed.ncbi.nlm.nih.gov/). The search used the terms “{[asthma (MeSH Terms)] AND [(hyperresponsiveness) OR (inflammation) OR (remodeling)]} AND [English (Language)]”, which were thoroughly reviewed manually by reading the full text or abstract carefully. We documented the risk biomolecules exactly implicated in airway inflammation, airway hyperresponsiveness, or airway remodeling in asthma. Only biomolecule sets supported by information from typical biological methods (Western blotting, PCR, ELISA, TaqMan assays, et al.) and statistical significance were considered and curated.

### 2.2 Go Ontology Analysis

To measure the functional similarities and differences in cellular components, biological process, and the molecular function of the disease feature-associated genes, GOSemSim (Yu et al., 2010), an R package for semantic similarity computation among GO terms, sets of GO terms, gene products and gene clusters, was applied.

### 2.3 MiRNA Data and miRNA Target Genes

Human miRNA information was obtained from miRBase v22 (http://www.mirbase.org/) (Kozomara et al., 2019). Human miRNA target data was acquired from DIANA-TarBase v8 (http://www.microrna.gr/tarbase/) (Karagkouni et al., 2018), DIANA-LncBase Experimental v2 (https://diana.e-ce.uth.gr/lncbasev3) (Paraskevopoulou et al., 2016), and miRTarBase v8 (http://miRTarBase.cuhk.edu.cn/) (Huang et al., 2020). Some were also derived from recently published research. (Supplementary Table S1).

### 2.4 MISIM-Based miRNA Functional Similarity Network and STRING-Based Protein–Protein Interaction Network

MISIM v2 (http://www.lirmed.com/misim/) (Li et al., 2019), a widely cited server for inferring miRNA functional similarity based on miRNA-disease associations and the expression level of miRNA, was applied to obtain miRNA-miRNA network weighted by functional similarity.

A synthetic network supported by six distinct evidence types was acquired from STRING v11 (https://string-db.org/) (Szklarczyk et al., 2019). These types comprised manually collected and consolidated protein-protein interactions, experimentally validated protein-protein interactions, and protein-protein interaction derived from an orthology-based model organism and computational association prediction based upon the whole-genome comparisons on fusion events, gene proximity, and expression profiles for each feature of asthma. We chose all of the STRING channels besides “text-mining” and adopted the Bayesian network approach provided by STRING for integration.

### 2.5 Pathway Collection

Pathway data was retrieved from several databases, including KEGG (updated on April 1, 2021, https://www.kegg.jp/) (Kanehisa and Goto, 2000), a database resource for understanding high-level functions and utilities of the biological system; Reactome (updated in September 2018, https://reactome.org/download-data/) (Fabregat et al., 2018), a free, open-source, curated and peer-reviewed pathway database; and the public Wikipathway (updated in September 2021, https://www.wikipathways.org) (Martens et al., 2021) resource.

### 2.6 Single Nucleotide Polymorphism Data Collection

The miRSNPs within miRNA target sites and miRNA gene were acquired in miRNASNP v3 (updated in 2020, http://bioinfo.life.hust.edu.cn/miRNASNP#!/) (Liu et al., 2021) and MSDD (updated in 2017) (Yue et al., 2018). The lncSNPs within lncRNA and the miRNA binding sites of lncRNAs were obtained from lncRNASNP2 (updated in 2018, http://bioinfo.life.hust.edu.cn/lncRNASNP#!/) (Miao et al., 2018) and LnCeVar (updated in 2020, http://www.bio-bigdata.net/LnCeVar/) (Wang et al., 2020).

### 2.7 MIMP Algorithm

In this study, MIMP was developed to prioritize the pathway through decoding the multiplex-heterogeneous network containing the mutual and internal relationships of mRNAs, miRNAs, and pathways. It proceeded in three steps: firstly, the multiplex-heterogeneous network was constructed and transformed into a commixture matrix. Secondly, random walk with a restart in the multiplex-heterogeneous network and singular value decomposition (SVD) was applied to the output. Thirdly, the relevance of mRNA’s and miRNA’s pathways were measured using the similarity between determined vectors in the same low-dimensional vector space with cosine similarity (Supplementary Material). The code sources and data were packaged into an R package called MIMP (which can be downloaded https://github.com/rmyhandsome/MIMP).

## 3 Results

### 3.1 Compiling the Asthma Risk Transcriptome Catalog

**FIGURE 1:**
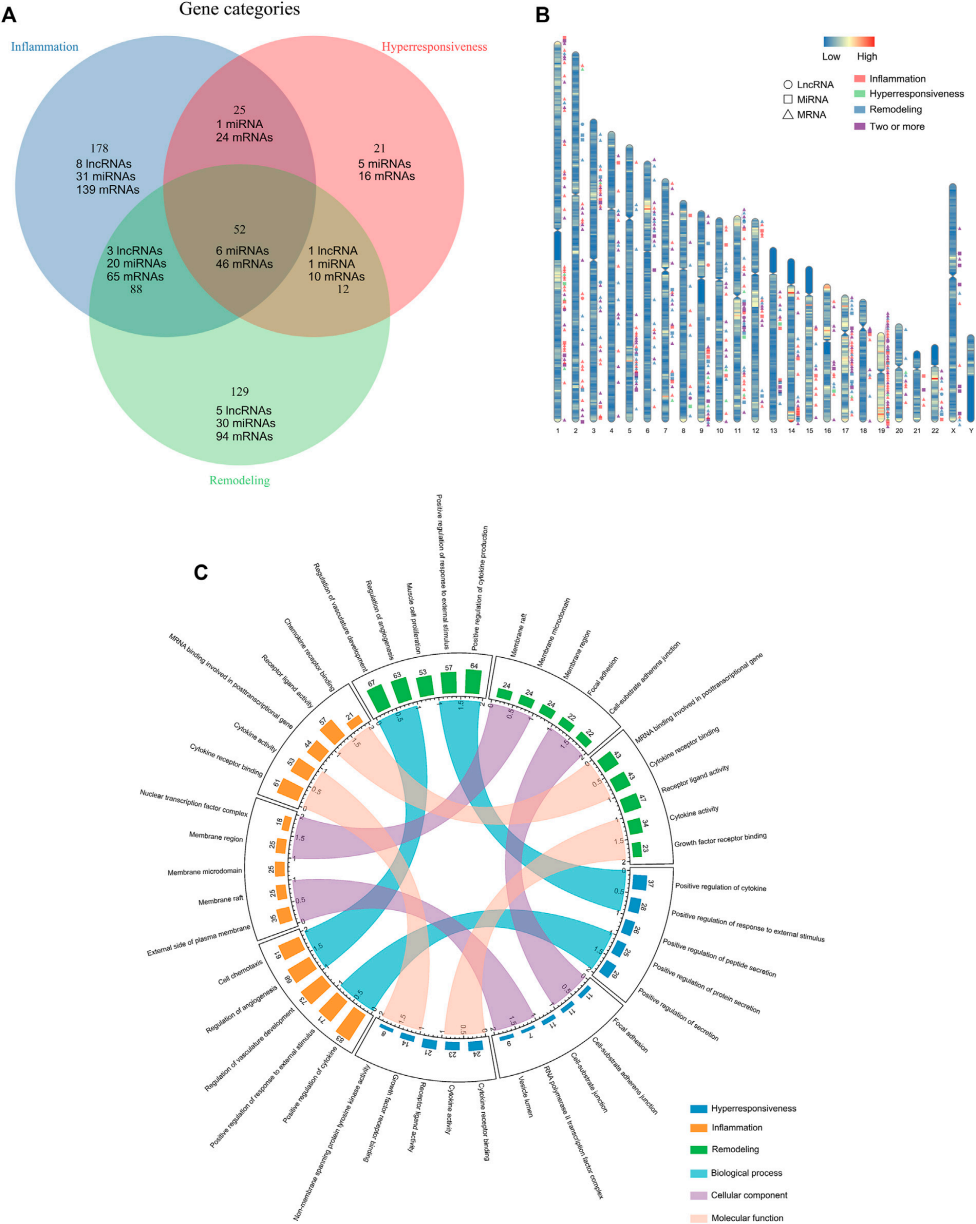
Compiling the disease feature risk transcriptome. **(A)** The distribution of disease characteristics-associated genes. **(B)** Spatial distribution of disease characteristics-related genes on chromosomes. **(C)** The top five statistically significant Go term annotations of disease characteristics-associated genes and the similarity measuring.

In total, 505 asthma risk biomolecules were identified by manual literature-mining, including 17 lncRNAs (two of them have not yet been validated in human tissues or cells), 94 miRNAs, 394 mRNAs (Figure 1A). We displayed their spatial distribution on chromosomes (Figure 1B). Interestingly, the risk transcriptome associated with the same disease characteristic is always inclined to clustering property, which may be the impetus of regulating disease progression. The go annotations applied to three disease characteristic-related gene sets revealed that the inflammation-associated genes were predominantly grouped into categories such as cytokine activity and chemokine receptor binding. The hyperresponsive-associated genes were mainly grouped into growth factor activity, protein tyrosine kinase activity, and NF-kappa-B binding. The remodeling-associated genes principally contained smooth muscle cell proliferation, cytokine activity, and growth factor receptor binding, in concert with current knowledge of asthma pathogenesis. Furthermore, we measured the similarity of three Go annotation sets in terms of biological process (BP), cellular components (CC), and molecular function (MF) with GoSemSim (Figure 1C). The details of these long tails in Figure 1C are shown in Supplementary Figure S1. The results revealed that inflammation-associated Go annotations were closer to remodeling-Go annotations than hyperresponsive-Go annotations at the level of BP, CC, and MF. However, we did not observe any particular differences, which may preliminarily indicate the existence of associations between the disease characteristics of asthma.

### 3.2 Pathway Prioritization in Disease Characteristics

**FIGURE 2:**
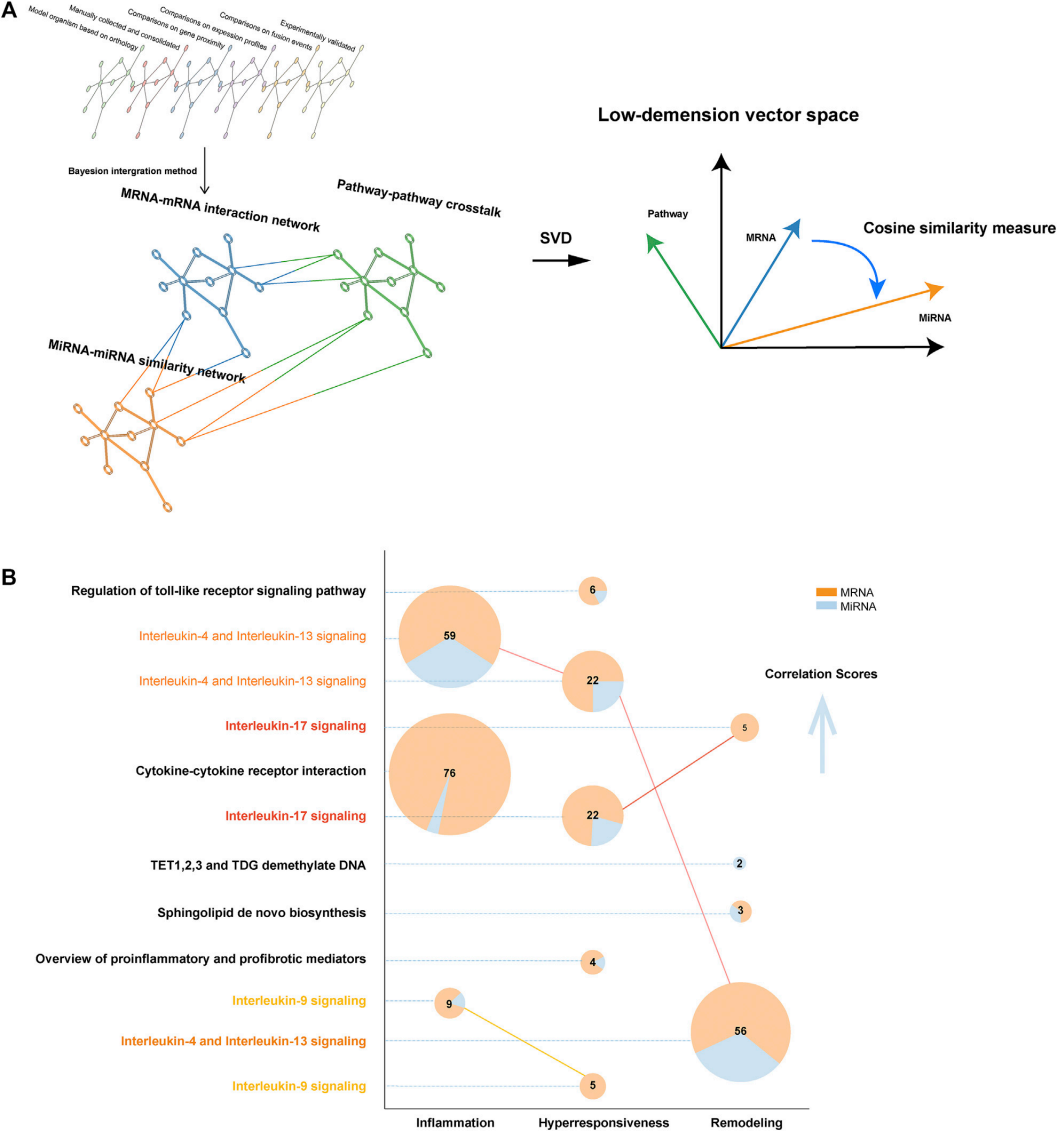
The structure of MIMP and pathway result exhibition. **(A)** The structure illustration of complex multiplex-heterogeneous network embedding in MIMP. **(B)** Robust risk pathways of disease characteristics. The number of genes mapping to pathways was directly labeled and the distribution of diverse gene types was color-coded. Pathways that were statistically significant in multiple disease features are highlighted via colored names and linked to the pie plot. The vertical axis from bottom to top shows the ordering of the empirical *p*-values from large to small, that statistical significance tends to increase.

We built a complex multiplex-heterogeneous network for each disease characteristic, encoding luxuriant biological information (Figure 2A). Relying on the output of this network structure, we calculated a correlation score for each pathway. Then ranking score was defined to correct the bias caused by pathway data set size derived from different databases. (Supplementary Material).

The present study aimed to locate the most significant disease feature pathway and thus explored the ceRNA-SNP-mediated dysfunction. We found that a ranking score of six is a significant indicator that can distinguish the high from the low score. In total, 12 pathways were eventually identified in asthma by MIMP, of which 3, 5, and 4 were associated with inflammation, hyperresponsiveness, and remodeling respectively (Figure 2B). The complete results are shown in Supplementary Table S2. There were multiple directionalities of the pathways, indicating that interleukin-17 signaling was concerned with both hyperresponsiveness and remodeling, interleukin-9 signaling was concerned with both inflammation and hyperresponsiveness, and interleukin-4 and Interleukin-13 signaled that all three features were related to the subtle difference of Go annotations of disease characteristics-related risk genes. Through mapping genes to pathways (Figure 2B), we correlated the scores of risk pathways that are usually irrelevant to overlapping parts. The pathways that overlapped with the feature risk genes did not show a better score than the identified feature pathways. Even when the same pathway was associated with different disease features, the larger overlapping part did not mean higher statistical significance. For example, interleukin-4 and Interleukin-13 signaling contained fewer overlapping genes but contrary to expectations, exhibited a higher statistical significance. The same phenomenon appeared in interleukin-17 signaling. We preliminarily concentrated on the effectiveness of statistically significant pathways with few overlapping genes. Some pathways had been reported and validated in previous studies such as interleukin-17 signaling and airway remodeling (overlapping mRNAs number is 5) (Evasovic and Singer, 2019; Camargo et al., 2020) and the regulation of sphingolipid (overlapping size is 2) to airway remodeling (Petrache and Petrusca, 2013; Yu et al., 2017). An individual pathway, TET 1,2,3 and TDG demethylate DNA (remodeling) had not been suggested to be associated with corresponding asthma characteristics before. Yet, the description of biological function and corresponding articles in the present study, indicated that it might relate to airway remodeling because of the quarried association between DNA methylation and airway remodeling (Clifford et al., 2012; Lin et al., 2016). Previous studies also revealed the difference in TET activity between nonasthmatic cells and asthmatic ASM cells (Somineni et al., 2016; Yeung et al., 2020).

In addition, we undertook enrichment analysis of feature risk genes using the traditional method based on the overlap among pathway members, the *p*-value was cut off by 0.05 (Raudvere et al., 2019). In total, 69 pathways were found in KEGG, 34 pathways in Reactome, and 80 pathways in Wikipathway. Then, 127 pathways in KEGG, 156 pathways in Reactome, and 181 pathways in Wikipathway, 114 pathways in KEGG, 105 pathways in Reactome, and 156 pathways in Wikipathway were found to be associated with hyperresponsiveness, remodeling, and inflammation respectively (see Supplementary Table S3). Such a huge quantity of statistically significant results made it hard to determine the center of research. Some notable phenomena were revealed when we compared them with former results from MIMP. These revealed 3, 4, and 2 pathways respectively that were concerned with inflammation (total 3), hyperresponsiveness (total 5), and remodeling (total 4) from MIMP results, which overlapped with the traditional results. The overlap occupied the relatively forward position of the ordered *p*-value of traditional results from small to large. The Sphingolipid *de novo* biosynthesis (remodeling), TET1,2,3, TDG demethylate DNA (remodeling), and Stimuli-sensing channels (hyperresponsiveness), which preliminarily revealed the non-negligible associations with their corresponding disease features, tend to be ignored in traditional gene pathway enrichment methods. However, in contrast to previous studies, our MIMP captured them. Combining pathways with genetic information, the Sphingolipid *de novo* biosynthesis also showed remarkable importance in the following analysis. To gather evidence relating to these results, we took the first ten pathway correlation scores from KEGG, Reactome, and Wikipathway, for each disease feature (total 90 pathways, all pathways were statistically significant) and compared them with the traditional pathway enrichment method. The large overlapping (64/90, approximately 71.1%) examples indicated that it was well-directed in detecting disease feature pathways. Even more remarkable is the fact that this was also the case for the specific pathways detected by MIMP. We searched PubMed for research on the relationship between asthma disease features and corresponding pathways (Supplementary Figure S2). The search revealed that only three pathways (3/26, approximately 11.5%) that corresponding publications did not been found but they were all possibly correlated with asthma after consideration of their biological functions. In the rest of the selected pathways, each pathway had at least four associated publications, exhibiting the reliability of the results produced by MIMP. This indicates that MIMP possesses great ability in excavating potential pathways with abundant and reliable results. It provided quantitative criteria for the distance of the disease and pathway rather than just generally observing whether the *p* value was less than 0.05/0.01 or not. MIMP reduced the resulting redundancy and overlapping-dependency on traditional pathway enrichment as part of the significance measure, which made it a preeminent supplementary method of examining extensive pathway enrichments. The MIMP algorithm could thus provide reliable and vital support for our future research.

### 3.3 Constructing ceRNA-Mediated SNP Switching Pathway Network

We generated a network-based dissection of the regulation to the pathway of ceRNA and ceRNASNPs. In total, 9 ceRNA regulatory networks composed of risk transcriptome were excavated from 7 risk pathways, of which 2, 4, and 3 were concerned with hyperresponsiveness, remodeling, and inflammation respectively. Then, we searched and exhibited the lncSNPs and miRSNPs in the ceRNA network while concentrating on three types (called regulatory SNPs), i.e., the 3′UTR, seed region of mature miRNA and miRNA binding site on lncRNA, which directly affects ceRNA regulation. We show the complete information of ceRNASNPs in Supplementary Table S4.

**FIGURE 3:**
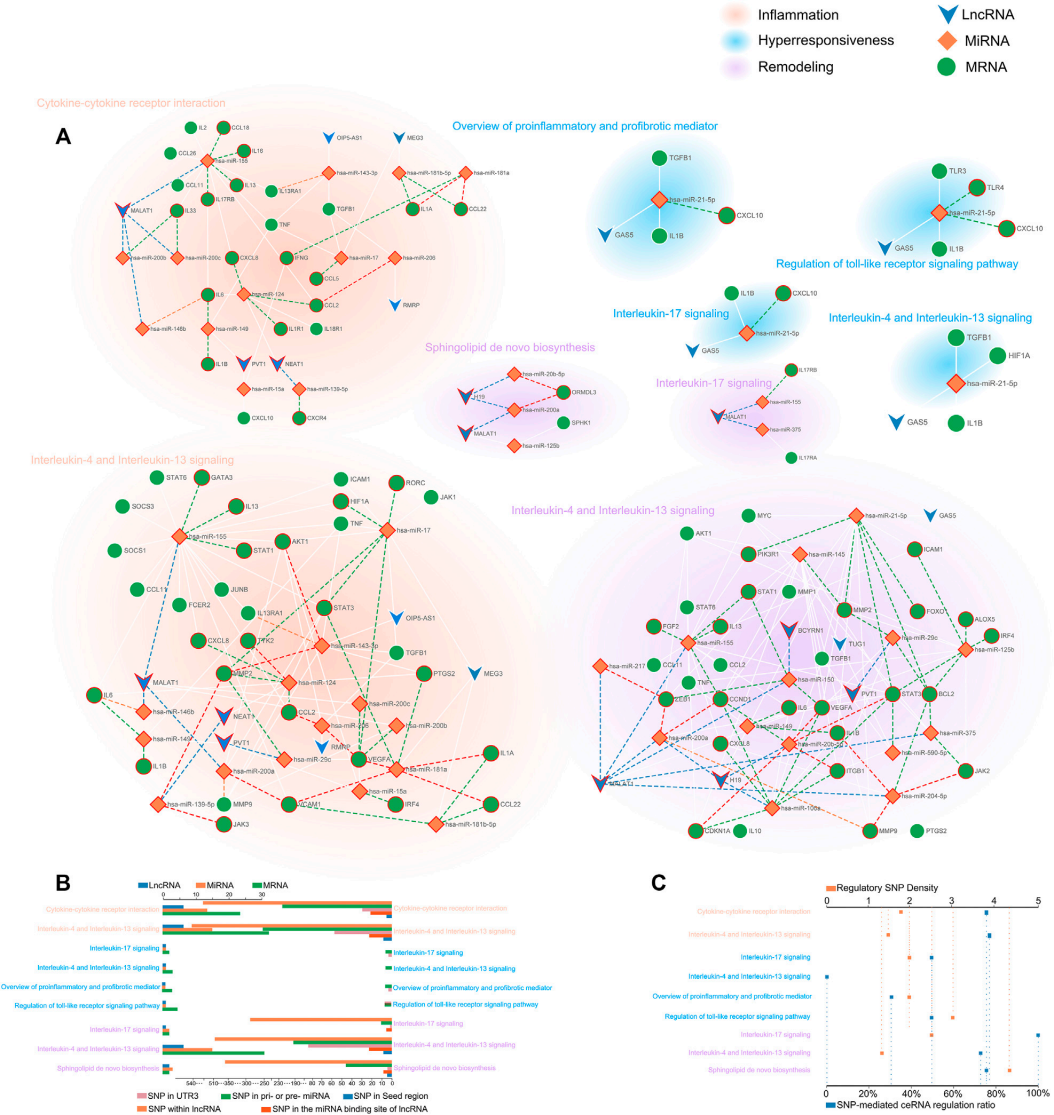
The comprehensive disease regulatory network and information decoding. **(A)** CSSPN. Green circle, orange rhombuses, and blue inverted triangles represent mRNAs, miRNAs, and lncRNAs respectively. The SNPs located in lncRNA, pri-/pre-miRNA, and the 3′UTR of mRNA were shown with the red circle around the mRNAs, miRNAs, and lncRNAs. The SNPs within the 3′UTR of mRNA, the seed region of mature miRNA, and the miRNA binding site on lncRNA were exhibited with green, orange, and blue dash lines respectively. The red dash line indicates the seed region of mature miRNA and 3′UTR of mRNA contain SNPs, which could affect miRNA-mRNA binding. **(B)** The multi-bar plot shows gene mapping and SNP mapping information pathways. The top and bottom axis represented the number of mapping genes and mapping SNPs respectively. **(C)** The dot plot of regulatory SNPs density and ceRNA regulation with SNP ratio. The top axis showed the density of regulatory SNPs. The percentage represented by the bottom axis reflects the proportion of ceRNA regulation with SNP in the total ceRNA regulations.

The CSSPN (ceRNA-mediated SNP switching pathway network) was constructed to elaborate on the regulatory role of ceRNA and the potential influence of SNPs on asthma features at the pathway level. Consequently, we obtained a comprehensive disease regulatory network partitioned for diverse disease features (Figure 3A). To gain detailed information on networks, we figured that the distribution of biomolecule types and SNP types (Figure 3B). A salient pathway, which is Interleukin-4 and Interleukin-13 signaling, possesses the largest proportion in risk biomolecules or SNPs in hyperresponsiveness, inflammation, and remodeling. This pathway has excellent potential as a bridge for these three disease characteristics. To measure the extent of the ceRNA network affected by regulatory SNPs, we further defined the average regulatory SNP density as the number ratio of regulatory SNPs to the total number of ceRNA regulations containing them in the network. We also calculated the proportion of ceRNA regulation containing regulatory SNPs in all ceRNA regulations (Figure 3C). As a result, a ceRNA network concerned with remodeling called “Sphingolipid *de novo* biosynthesis” possessed the largest average of regulatory SNP density. The highest SNP-mediated ceRNA regulation ratio belonged to Interleukin-17 signaling (remodeling), followed by the Interleukin-4 and interleukin-13 signaling and Sphingolipid *de novo* biosynthesis (over 75%).

### 3.4 Dissection of Potential Mechanisms of Polymorphic “Switch” Influencing ceRNA Regulation to Asthma Risk Pathway

#### 3.4.1 Comprehensive Feature Pathway Map of Asthma

**FIGURE 4:**
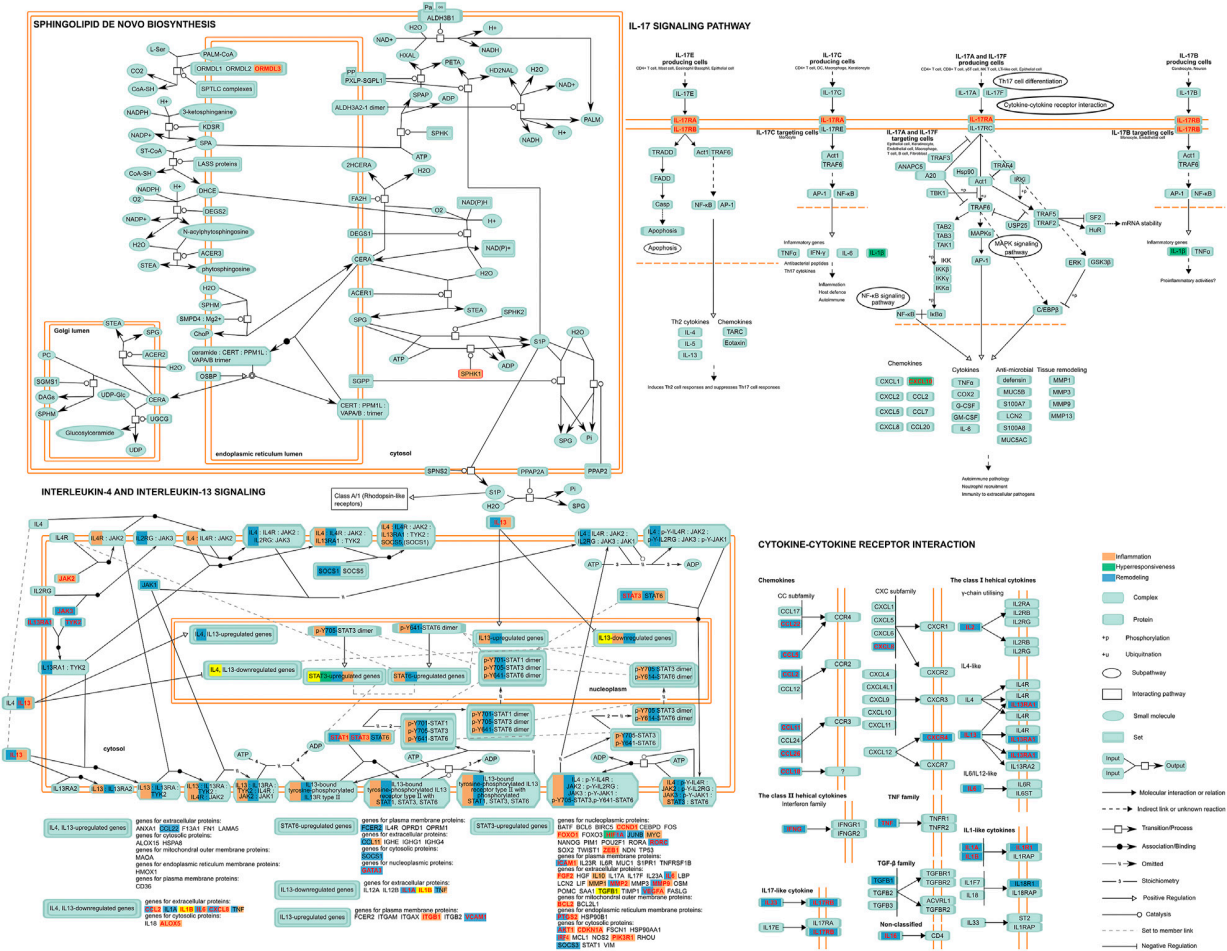
A depiction of the disease features of the ceRNA network-involved pathway. Proteins encoded by risk genes concerning inflammation, hyperresponsiveness, and remodeling and related complexes are indicated in orange, green, and blue respectively, while those in red character represented encoding genes indicate the ceRNA regulation-containing genes with regulatory SNPs. Yellow represents the significant elements related to all disease features in a pathway.

Although there has been a large amount of research conducted on SNPs embedding in disease feature ceRNA networks to date, the significant role of SNPs in biological progress needs to be combined with pathway data for analysis. Thus, we carried out an in-depth dissection of these crucial pathways and identified the location of those genes of the classified ceRNA network in the pathway map (Figure 4). This consequently revealed that some risk genes occupy crucial places located on the upstream pathways, controlling origination of the whole pathway regulation as “switch”. For instance, for IL-17RA and IL-17RB (remodeling) in the Interleukin-17 signaling pathway, encoding the receptor proteins located on the cytomembrance is the first stage of entering into the cell to play its biological role in IL-17 family cytokines. In other words, the regulatory SNPs concerning these genes could directly affect the whole function of pathways.

#### 3.4.2 The Regulation of Regulatory SNP of Focused ceRNA Over the Pathway

**FIGURE 5:**
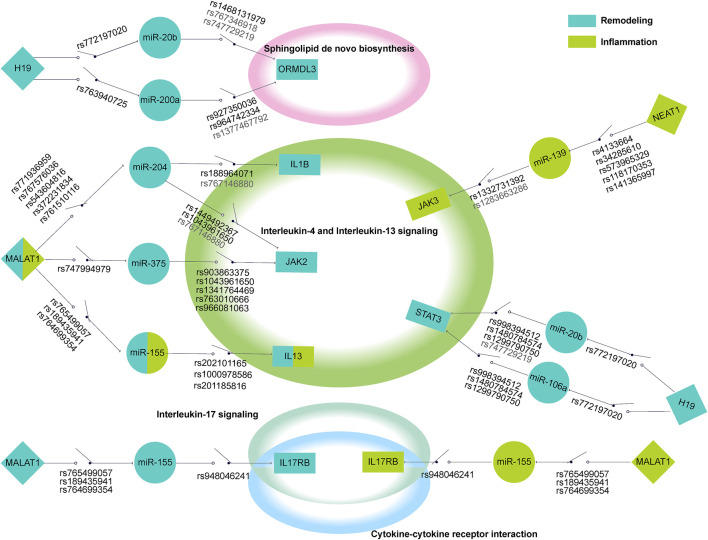
The schematic diagram of regulatory SNPs → gene → pathway effect of focused genes. The green and blue represented the genes in the risk pathways concerning inflammation and remodeling respectively. The diamond, circle, and rectangle represent lncRNA, miRNA, and mRNA respectively. The line between them shows the regulation of ceRNA. The “on-off” symbol sited on line between lncRNA and miRNA or miRNA and mRNA indicate the regulatory SNP within the miRNA binding site on lncRNA or within the mature miRNA seed region (character colored in deep grey) or the 3′UTR region (character colored in black). The peripheral large circles or rectangles denoted the pathways that regulatory SNPs may influence through their effects on ceRNA regulations to target genes.

According to the characteristics of gene distribution in the comprehensive pathway diagram mentioned above, we thoroughly searched for the genes simultaneously obeying the property of “switch” and containing the regulatory SNPs on corresponding ceRNA regulations. ORMDL3 (remodeling) of Sphingolipid *de novo* biosynthesis, JAK2 (remodeling), JAK3, IL13RA1, TYK2 (inflammation), and IL13, STAT3 (inflammation/remodeling) of Interleukin-4 and Interleukin-13 signaling, IL17RA of Interleukin-17 signaling, and IL17RB of both Cytokine-cytokine receptor interaction and Interleukin-17 signaling were finally locked. Two genes, CXCL10 (hyperresponsiveness) of Interleukin-17 signaling and IL1B (inflammation/remodeling/hyperresponsiveness) of Interleukin-4 and Interleukin-13 signaling were taken into consideration because the former was the only gene containing regulatory SNPs on ceRNA regulation in the hyperresponsiveness-associated network and the other one correlated with all disease features. We selected the ceRNA regulations of these genes, which contained regulatory SNPs both on lncRNA-miRNA and miRNA-mRNA regulation, and characteristically visualized them (Figure 5). We finally discovered 11 ceRNA regulations, which contained eight disease risk genes of four pathways involving two disease features, i.e., inflammation and remodeling. We noticed two arresting ceRNA regulations. As described above, Interleukin-4 and Interleukin-13 signaling is simultaneously associated with hyperresponsiveness, inflammation, and remodeling. After searching this pathway, we found that MALAT1 → hsa-miR-155 → IL13 participated in both remodeling and inflammation. The MALAT1 → hsa-miR-155 → IL17RB was discovered as an overlapping of two different disease feature pathways, Interleukin-17 signaling (remodeling) and Cytokine-cytokine receptor interaction (inflammation). The significant role of MALAT1 and miR-155 in asthma has been universally demonstrated. Overall, they could alter the Th1/Th2 balance of asthma and induce proliferation and migration of airway smooth muscle cells (Shi et al., 2018; Lin et al., 2019; Liang and Tang, 2020). Earlier research also revealed the expression dysregulation of miR-155 (Karam and Abd Elrahman, 2019). IL-17 and IL-13, two types of interleukin family, are common research subjects in the pathogenesis of asthma and are often studied as therapeutic targets for asthma (Ntontsi et al., 2018; Ramakrishnan et al., 2019). They also occupied vital locations in their pathway, which almost controlled the whole pathway as a “switch”.

**FIGURE 6:**
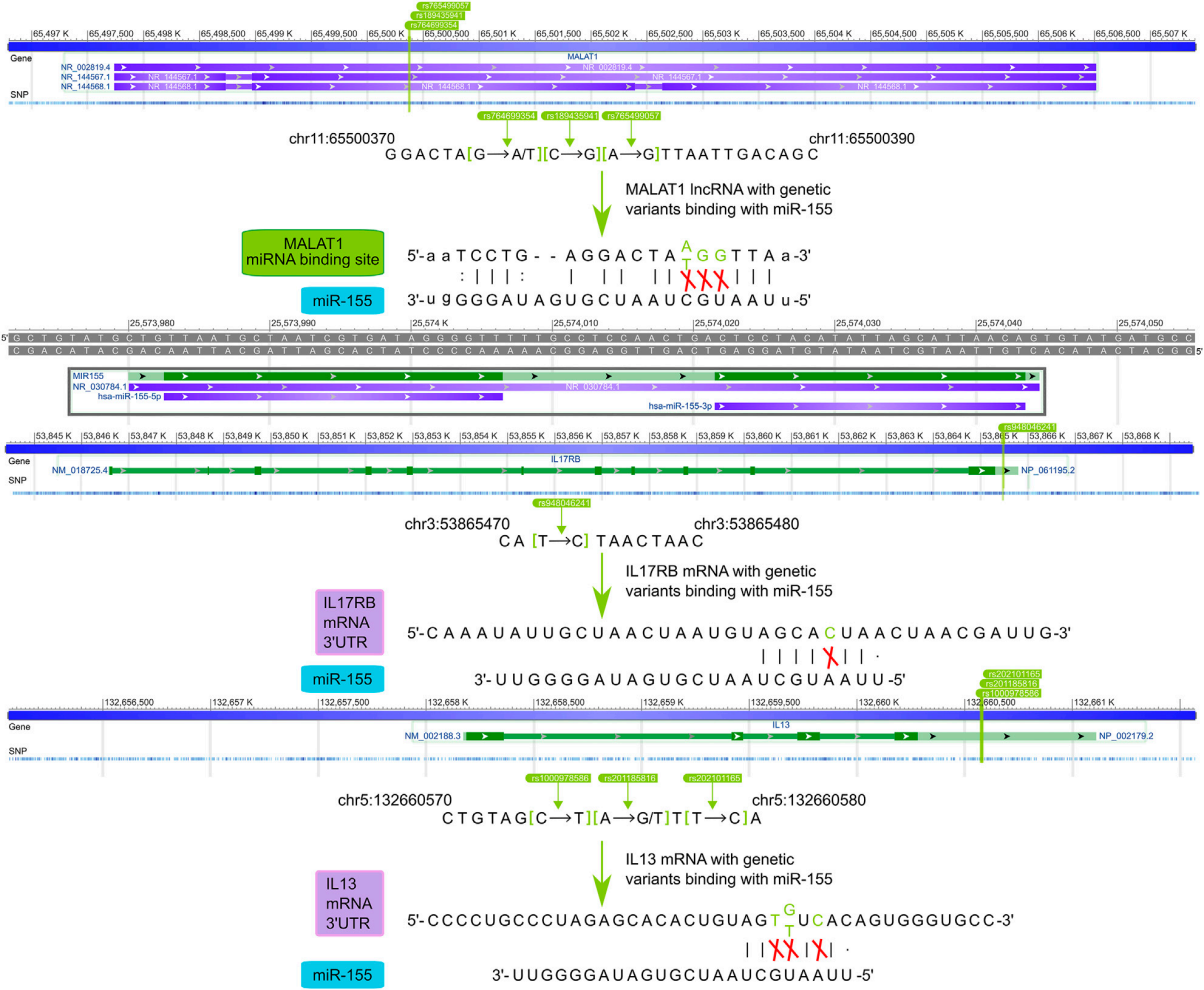
The potential mechanisms of regulatory SNPs influence IL13 and IL17RB *via* ceRNA regulation.

We further exhibited how the regulatory SNPs altered the balanced regulation of ceRNA, inspecting the potential molecular mechanism involved in the characteristic pathways of asthma diseases. (Figure 6). Our analysis showed the crucial influence of rs765499057/rs764699354/rs189435941 on the miRNA binding site of lncRNA MALAT1→hsa-miR-155 and rs201185816/rs1000978586/rs202101165 on the 3′UTR of the IL13 regulated Interleukin-4 and Interleukin-13 signaling pathway via destroying or weakening ceRNA regulations. Another biological pathway, Interleukin-17 signaling was revealed to also be under the impact of regulatory SNPs, i.e., rs765499057/rs764699354/rs189435941 on the miRNA binding site of lncRNA MALAT1→hsa-miR-155 and rs948046241 on the 3′UTR of IL17RB. The momentous significance of these two disease feature pathways in asthma has been widely discussed above. The regulatory SNPs we have excavated could play a crucial role in asthma by disturbing the balance of ceRNA regulation.

## 4 Discussion

The dissection of regulatory SNPs in ceRNA regulation would help to elucidate their potential roles in asthma pathogenesis both as genetic variants and at the post-transcriptional regulation level. In this study, we identified asthma disease feature pathways with a novel algorithm called MIMP for the first time. We systematically excavated candidate functional regulatory SNPs in the associated ceRNA regulations and their potential mechanisms of switching the pathways based on current genetic findings. We manually compiled the comprehensive catalog of asthma disease feature transcriptome, which was categorized into three types, inflammation, airway hyperresponsiveness, and airway remodeling. The similarity of Go term annotation was measured by a common graphic method. Since current traditional gene pathway enrichment methods could only make fractional use of the genetic information obtained from our text mining, we developed a novel algorithm based on a complex heterogeneous network for excavating pathways via decoding the mutual and internal relationships of mRNA, miRNA, and pathways. We next established the ceRNA network of each pathway with the risk transcriptome and experimentally supported miRNA-target information. By constructing CSSPN, searching and screening reliable database information, we have further revealed the candidate functional regulatory SNP “switches” in ceRNA regulation that regulate the disease feature pathways of asthma. Furthermore, several significant genes at the crucial location of disease feature pathways were confirmed. We filtered the curated ceRNA regulations related to these elite genes, which contain regulatory SNPs on the binding sites of both lncRNA-miRNA and miRNA-lncRNA, and then mapped them to the asthma feature pathways. In addition, we carried out an in-depth dissection of the correlation between these ceRNA regulations and mapped pathways, elaborated the significance of two high-risk ceRNA regulations, and proposed the potential mechanisms of particular regulatory SNPs as “switches” in ceRNA regulation of the asthma disease feature pathways.

Our disease feature pathway analysis simultaneously captured the specific pathway regulation of individual features and the overlap of pathway regulation of diverse disease features, thus expounding the association of asthma in terms of biological pathways. Upregulation of ORMDL3 disrupts homeostatic levels of ceramides and sphingolipid metabolite within the endoplasmic reticulum, later promoting airway smooth muscle remodeling (James et al., 2019). Our analysis and database mining revealed that ORMDL3 played a key role as a “switch” to the airway remodeling risk pathway of Sphingolipid *de novo* biosynthesis and contained regulatory SNPs which might influence its functions via breaking the related ceRNA regulation, i.e., H19 → miR-20b/miR-200a → ORMDL3. rs772197020 and rs763940725 were the genetic variants in the binding site of lncRNA H19-hsa-miR-20b-5p and lncRNA H19-hsa-miR-200a. A previous study has reported the expression dysregulation of miR-20b/miR-200a in asthma airway remodeling (Tang and Luo, 2018; Zhu et al., 2020a). rs1468131979 in 3′UTR and rs767346918/rs747729219 in the seed region of miR-20b, rs927350036/rs96742334 in 3′UTR and rs1377467792 in seed region of miR-200a could naturally disturb the normal biological function of ORMDL3 by affecting the biogenesis of ORMDL3. The accumulated analysis supports the potential mechanism, suggesting regulatory SNPs → ORMDL3 → Sphingolipid *de novo* biosynthesis → airway remodeling in asthma. We also captured some significant overlaps of asthma disease features in the terms of pathways, of which the overlapping regulation of inflammation and airway remodeling in Interleukin-4 and Interleukin-13 signaling was especially elaborated. The association between the two disease features of asthma from a clinical perspective has been noticed in previous research (Holgate, 2002; Zhou-Suckow et al., 2017; Banno et al., 2020). Our findings could provide support for the further study of these perspectives as a media connecting the clinical features with the molecular mechanism.

There were a total 182 regulatory SNP-mediated ceRNA regulations in the asthma disease feature pathways, of which 87, 10, and 85 were concerned with inflammation, airway hyperresponsiveness, and airway remodeling respectively. Some of them have been reported to be significantly relevant to asthma such as NEAT1 → hsa-miR-139→ JAK3 (Zhu et al., 2021). According to the “switch” role of elite genes to their pathways, 11 crucial ceRNA regulations were naturally captured, of which 2, i.e., MALAT1 (rs765499057/rs764699354/rs189435941) → hsa-miR-155 → IL13 (rs201185816/rs1000978586/rs202101165) → Interleukin-4 and Interleukin-13 signaling → inflammation/airway remodeling and MALAT1 (rs765499057/rs764699354/rs189435941) → hsa-miR-155 → IL17RB (rs948046241) → Interleukin-17 signaling (airway remodeling)/Cytokine-cytokine receptor interaction (inflammation) were further explored, examining the disturbance of regulatory SNPs in the pathway in terms of molecular mechanism. Although the effective excavation of asthma disease feature pathways in the early stages of our work was based on experimentally supported biological information, these potential mechanisms should be interpreted with caution due to the temporary lack of definite experimental evidence. The MIMP was also limited by incomplete data, which has been a universal objective reason beyond control, including disease-associated transcriptome, miRNA-mRNA, and lncRNA-miRNA regulation and pathway completeness. More valuable work can be progressed in the future. Gene expression patterns in a specific disease may be integrated into the mRNA-mRNA similarity network. Transcription factor regulatory network and lncRNA regulation could also contribute to excavating potential disease risk pathways. We will also focus on the downstream dysfunction mediated by SNP located in transcription factors, which could be comprehensively analyzed through but not limited to the network method. Nonetheless, our approach has created a new perspective to explore the pathogenesis of asthma, capturing diverse characteristics that divide the whole into different main parts for more delicate observation at the level of the pathway, contributing to capturing the primary and specific parts.

In addition to serving the asthmatic signature pathway mining, our novel algorithm MIMP could be applied with enrichment pathway prioritization for any characteristic sets consisting of miRNAs and mRNAs. It showed an impressive ability to reduce the resulting redundancy and overlapping-dependency of the traditional pathway enrichment method while keeping the result in a strong connection with known biological information and developing the continuous renewal of the biological information demanded. The algorithm is recommended as a widely applicable auxiliary supplement to gene pathway enrichment methods, contributing to the deep excavation of potential pathways. Our research shows the potential to contribute to future experimental studies of ceRNA regulation or SNPs in asthma, especially in consideration of the lack of exploration in this field at present.

## Data Availability

The datasets presented in this study can be found in online repositories. The names of the repository/repositories and accession number(s) can be found in the article/Supplementary Material.
