# Supplementary figures and images for: Multiplex-Heterogeneous Network-Based Capturing Potential SNP “Switches” of Pathways Associating With Diverse Disease Characteristics of Asthma
Source: Front Cell Dev Biol. 2021 Dec 14;9:744932. doi: 10.3389/fcell.2021.744932 (PMC8712737; doi:10.3389/fcell.2021.744932)

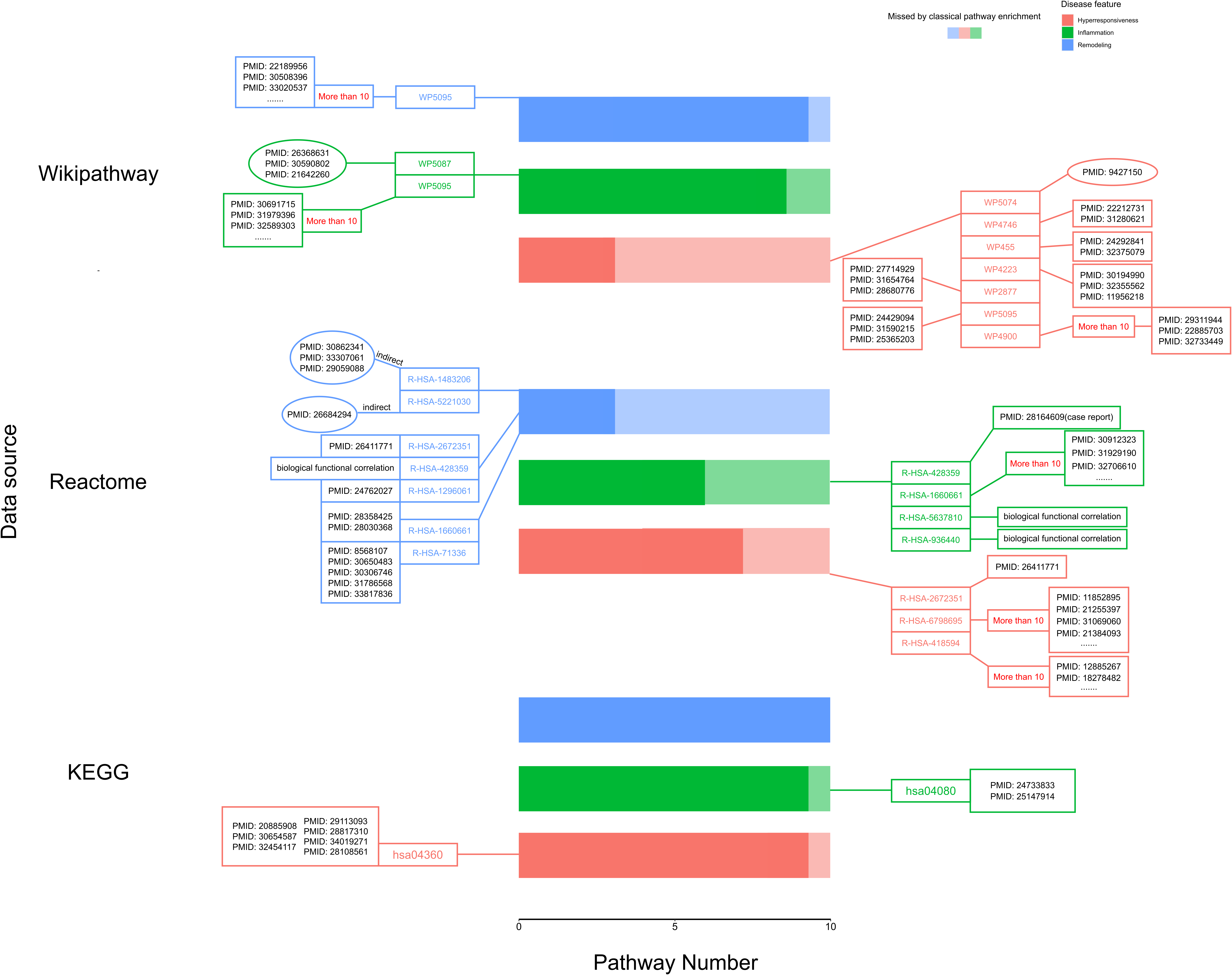

Supplement: Supplementary file 3 [file Image2.TIF]

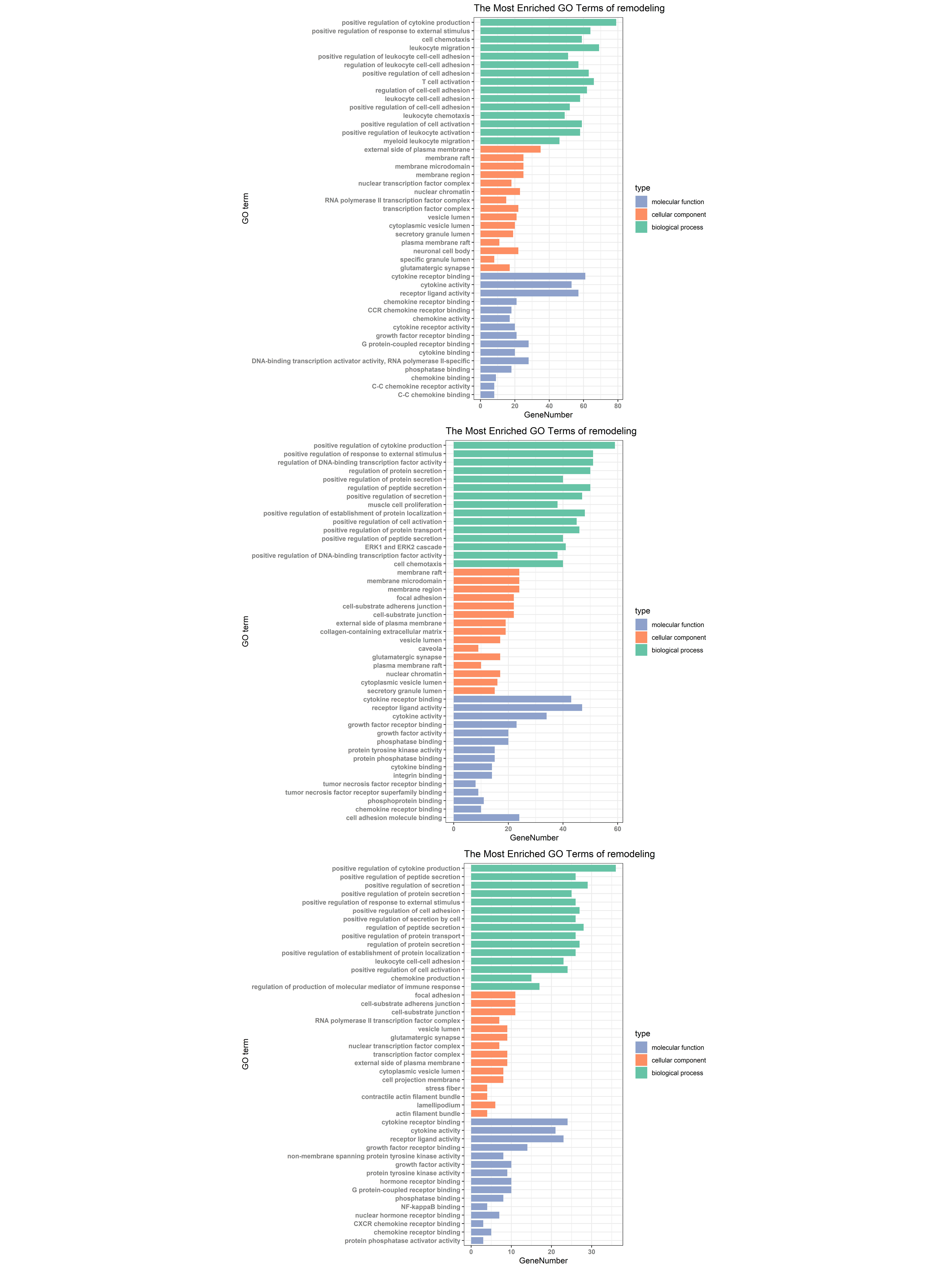

Supplement: Supplementary file 4 [file Image1.TIF]
