# Supplementary material for: Multiplex-Heterogeneous Network-Based Capturing Potential SNP “Switches” of Pathways Associating With Diverse Disease Characteristics of Asthma
Source: Front Cell Dev Biol. 2021 Dec 14;9:744932. doi: 10.3389/fcell.2021.744932 (PMC8712737; doi:10.3389/fcell.2021.744932)
Supplement: Supplementary file 5 [file DataSheet1.PDF]

MIMP algorithm

In this study, MIMP was developed to prioritize the pathway through decoding the multiplex-heterogeneous network which contained the mutual and internal relationships of mRNAs, miRNAs, and pathways. It proceeded in three steps: first, constructing the multiplex-heterogeneous network and transforming into a commixture matrix, secondly, random walking with restart in the multiplex-heterogeneous network and applying SVD on the output, thirdly, measuring the relevance of mRNA and miRNA to pathway by the similarity between determined vectors of them in the same low-dimensional vector space with cosine similarity.

Step 1: A heterogeneous network consist of mRNAs, miRNAs and pathways was established for integrating the STRING-based protein–protein interaction network, the MISIM-based miRNA functional similarity network and pathways information depicted above. A connection existed between two genes if they were interlinked in the network. A connection existed between two miRNAs if they were interlinked in the network. If miRNA regulated the gene, then connect them. An edge existed between gene and pathway if the pathway contained the gene. Linked miRNAs to pathways if they were correlative. Linked pathways to pathways if they were significantly crosslinking. Crosstalk among pathways and the correlation between miRNA and individual pathway in non-random data were identified by cumulative hypergeometric distribution. The formula was as follows:

$$p = F(x|M,J,N) = \sum_{i=0}^{x} \frac{\binom{J}{i}\binom{M-J}{N-i}}{\binom{M}{N}}$$

When identifying miRNA targeting pathways, M denoted the number of whole

human genome genes, J represented the number of genes in a given pathway, N denoted the number of target genes of a given miRNA, and x denoted the number of target genes involved in the pathway. Analogously, for analysis of pathway crosstalk, M denoted the number of whole human genome genes, J and N represented the number of genes in the two given pathways respectively, and x denoted the number of genes overlapping between the two given pathways. We adjusted p-values by the Benjamini and Hochberg false discovery rate to judge the statistical significance and we regarded two pathways as significantly crosslinking or a miRNA as targeting a pathway if the FDR < 0.05.

Formally, let P represented adjacency matrix of MISIM-based miRNA functional similarity network with *m* miRNAs. Let L represented adjacency matrix of STRING-based protein–protein interaction network with *n* genes. Let k represented the number of pathways. Let $G \in \{0,1\}^{m\times n}$ denoted the miRNA-gene association matrix, where $G_{ij} = 1$ if miRNA i targeted gene j. Let $E \in \{0,1\}^{n\times k}$ denoted the gene-pathway association matrix, where $E_{ij} = 1$ if gene i belonged to pathway j. Let $Q \in \{0,1\}^{m\times k}$ represented the miRNA-pathway association matrix, where $Q_{ij} = 1$ if miRNA i targeted pathway j. Let $S \in \{0,1\}^{n\times n}$ represented the pathways crosstalk matrix, where $\mathrm{S}_{ij} = 1$ if pathway i crosslinked pathway j .The multiplex-heterogeneous network $U \in \mathbb{R}^{(m+n+k)\times(m+n+k)}$ was then defined as:

$$
H_{ij} = \begin{cases} P_{ij}, & i \le m, j \le m \\ G_{ij}, & m < i \le m+n, j \le m \\ G_{ij}^{T}, & i \le m, m < j \le m+n \\ L_{ij}, & m < i \le m+n, m < j \le m+n \\ Q_{ij}^{T}, & i > m+n, j \le m \\ E_{ij}^{T}, & i > m+n, m < j \le m+n \\ Q_{ij}, & i \le m, j > m+n \\ E_{ij}, & m < i \le m+n, j > m+n \\ S_{ij}, & i > m+n, j > m+n \end{cases}
$$

Step2: RWR-MH (Random Walk with Restarts on Multiplex-Heterogeneous Network), a recently developed preferable method on multiplex-heterogeneous network representation, was embedded in the multiplex-heterogeneous network.

In the new RWR-MH algorithm, particle kept exploring the different network layers owing to the jumps and still leveraged the complementary biological information while remaining stability. Therefore, it had far better prioritization results than those of all other versions of the algorithm when applying on complex multiplex-heterogeneous network(Valdeolivas *et al.*, 2019). Because of these advantages to handle noisy and missing edges in the complex multiplex-heterogeneous biological network, RWR-MH had achieved state-of-the-art results in several computational biology tasks. (Fan *et al.*, 2019)

It outputted a matrix $W \in \mathbb{R}^{(m+n+k)\times(m+n+k)}$ which incorporates the decoding information of the complex multiplex-heterogeneous network. SVD (singular value decomposition) was the most popular matrix factorization method in the recommendation system for information filtering and enriching. It had been extensively applied in the dimension reduction of data in previous studies (Wu *et al.*, 2019; Zeng *et al.*, 2020). We imported it to capture low-dimensional vectors of miRNA, mRNA, and

pathway.

SVD took a square matrix $W \in \mathbb{R}^{(m+n+k)\times(m+n+k)}$. The SVD theorem stated:

$$W_{(m+n+k)\times(m+n+k)} = U_{(m+n+k)\times(m+n+k)}\Sigma_{(m+n+k)\times(m+n+k)}V^T_{(m+n+k)\times(m+n+k)}$$

Where the $U^T U = I_{(m+n+k)\times(m+n+k)}$ and $V^T V = I_{(m+n+k)\times(m+n+k)}$(i.e. U and V are orthogonal).

Where the columns of U were the left singular vectors; $\sum$ (the same dimensions as W) had singular values and was diagonal; and V^T had rows that were the right singular vectors. The SVD represented an expansion of the original data in a coordinate system where the covariance matrix was diagonal.

Calculating the SVD comprised finding the eigenvalues and eigenvectors of WW^T and $W^T W$. The eigenvectors of WW^T composed the columns of U, the eigenvectors of $W^T W$ composed the columns of V. Also, the singular values in $\sum$ were square roots of eigenvalues from WW^T or $W^T W$. The singular values were the diagonal entries of the $\sum$ matrix and were arranged in descending order. The singular values were always real numbers.

Dimension reduction with SVD was accomplished by defining an empirical parameter $\mathrm{r} < (m+n+k)$ to acquire $W_{r\times(m+n+k)}$ which contained the main information of the matrix(Cho *et al.*, 2016) :

$$W_{r\times(m+n+k)} = \textstyle\sum_{r\times r} V^T_{r\times(m+n+k)}$$

Step3: To measure the similarity between miRNA, mRNA and pathway, we adopted a classic and reliable standard namely the cosine similarity of the low-dimensional representations of them. Formally, the MIMP ranking score between

pathway i and disease characteristic j was defined as:

$$S_{ij} = \sum_{m \in DCmiRNAs(j), n \in DCmRNAs(j)} \cos(V_i, V_m) + \cos(V_i, V_n)$$

Where the $DCmiRNAs(j)$ and $DCmRNAs(j)$ represented the miRNAs set and the mRNAs set of disease characteristic j respectively.

We finally obtained the correlation scores of pathway and disease features. For each pathway, we calculated a ranking score describing the extent of bias from average value, formulating as $RS_i = \frac{CS_i - \mu_{CS}}{\sigma_{cs}}$, where the CS_i represented the correlation score of pathway i , the μ_{CS} and σ_{CS} represented the CS average value and standard deviation value of all detected pathways in certain disease feature. Thus RS was positively correlated with importance. Besides, for each disease feature, we randomly generated a new set of m miRNAs and n mRNAs with the same size of the contemporary catalog of risk transcriptome. Then we constructed a random multiplex-heterogeneous network with the same structure as above and the MIMP was embedded in. This was repeated $a = 1000$ times. With k pathways, total of ka random MIMP scores were ultimately produced. We measured the statistical significance for each selected high RS pathway by a typical statistical indicator, z-score, i.e. checking the position of real score in the distribution of random data (Note that the distribution of original or converted random data should satisfy the normality when using small data set size.).

References

Cho, H. *et al.* (2016) Compact Integration of Multi-Network Topology for Functional Analysis of Genes. *Cell systems*, **3**, 540-548.e5.

Fan, X.-N. *et al.* (2019) Prediction of lncRNA-disease associations by integrating diverse heterogeneous information sources with RWR algorithm and positive pointwise mutual information. *BMC bioinformatics*, **20**, 87.

Valdeolivas, A. *et al.* (2019) Random walk with restart on multiplex and heterogeneous biological networks. *Bioinformatics (Oxford, England)*, **35**, 497–505.

Wu, G. *et al.* (2019) Prediction of drug-disease associations based on ensemble meta paths and singular value decomposition. *BMC bioinformatics*, **20**, 134.

Zeng, M. *et al.* (2020) SDLDA: lncRNA-disease association prediction based on singular value decomposition and deep learning. *Methods (San Diego, Calif.)*, **179**, 73–80.
